# A common polymorphism in *NR1H2 *(LXRbeta) is associated with preeclampsia

**DOI:** 10.1186/1471-2350-12-145

**Published:** 2011-10-26

**Authors:** Kevin Mouzat, Eric Mercier, Anne Polge, Alexandre Evrard, Silvère Baron, Jean-Pierre Balducchi, Jean-Paul Brouillet, Serge Lumbroso, Jean-Christophe Gris

**Affiliations:** 1 Department of Biochemistry, Nimes University Hospital, F-30029 Nîmes Cedex 9, France; 2 Department of Hematology, Nimes University Hospital, F-30029 Nîmes Cedex 9, France; 3 GReD Laboratory, UMR CNRS 6247 - Clermont University - INSERM U931, F-63177 Aubière Cedex, France; 4 Department of Internal Medicine, Nimes University Hospital, F-30029 Nîmes Cedex 9, France

## Abstract

**Background:**

Preeclampsia is a frequent complication of pregnancy and a leading cause of perinatal mortality. Both genetic and environmental risk factors have been identified. Lipid metabolism, particularly cholesterol metabolism, is associated with this disease. Liver X receptors alpha (NR1H3, also known as LXRalpha) and beta (NR1H2, also known as LXRbeta) play a key role in lipid metabolism. They belong to the nuclear receptor superfamily and are activated by cholesterol derivatives. They have been implicated in preeclampsia because they modulate trophoblast invasion and regulate the expression of the endoglin (CD105) gene, a marker of preeclampsia. The aim of this study was to investigate associations between the *NR1H3 *and *NR1H2 *genes and preeclampsia.

**Methods:**

We assessed associations between single nucleotide polymorphisms of *NR1H3 *(rs2279238 and rs7120118) and *NR1H2 *(rs35463555 and rs2695121) and the disease in 155 individuals with preeclampsia and 305 controls. Genotypes were determined by high-resolution melting analysis. We then used a logistic regression model to analyze the different alleles and genotypes for those polymorphisms as a function of case/control status.

**Results:**

We found no association between *NR1H3 *SNPs and the disease, but the *NR1H2 *polymorphism rs2695121 was found to be strongly associated with preeclampsia (genotype C/C: adjusted odds ratio, 2.05; 95% CI, 1.04-4.05; *p *= 0.039 and genotype T/C: adjusted odds ratio, 1.85; 95% CI, 1.01-3.42; *p *= 0.049).

**Conclusions:**

This study provides the first evidence of an association between the *NR1H2 *gene and preeclampsia, adding to our understanding of the links between cholesterol metabolism and this disease.

## Background

Preeclampsia (PE) is a frequent complication of the second half of pregnancy and is one of the leading causes of maternal perinatal mortality and morbidity [1]. This condition, affecting 2.5 to 3% of pregnant women is defined by gestational hypertension accompanied by proteinuria [2,3]. Many risk factors have been described, including, in particular, cardiovascular risks, such as diabetes mellitus and high body mass index [4,5]. Cholesterol metabolism may also contribute to the pathogenesis of preeclampsia. Indeed, high total cholesterol and LDL (low density lipoprotein)-cholesterol levels are significantly associated with this disease [6]. Moreover, it has been shown that a familial history of PE almost triples the risk of a woman developing this disease, consistent with the involvement of genetic factors [4]. Many risk factors have been described, but the molecular mechanisms underlying this disease remain unclear. Liver X receptors (LXRs) NR1H3 and NR1H2, more commonly known as LXRalpha and LXRbeta, play a key role in cholesterol metabolism [7,8]. They belong to a subclass of nuclear receptors that form obligate heterodimers with 9-*cis *retinoic acid receptors (RXR). They also bind to and are activated by naturally occurring oxidized forms of cholesterol, known as oxysterols, the intracellular concentrations of which are directly correlated with cholesterol concentration [9]. Knockout mice deficient in oxysterol synthesis pathways are unable to induce LXR-target genes in response to dietary cholesterol, demonstrating that these nuclear receptors are endogenous receptors for oxysterols [10]. Upon ligand binding, they activate the transcription of many genes involved essentially in lipid metabolism, cholesterol metabolism in particular. Thus, by stimulating the cellular efflux and hepatic reverse transport of cholesterol and inhibiting its endogenous synthesis, they act as hypocholesterolemic factors [11]. LXRs may therefore be considered endogenous cholesterol sensors.

LXRs are promising candidates in investigations of the molecular causes of PE. It is now widely accepted that PE results from placentation defects. Indeed, any defect in trophoblast development or differentiation can result in PE [12]. LXRs are expressed in human placenta at various stages of pregnancy, right until term [13], and their expression increases during pregnancy [14]. In addition to their potential role in regulating placental lipid metabolism, LXRbeta has recently been shown to control trophoblast invasion *in vitro *[15]. Furthermore, we have also shown that *ENG*, encoding endoglin (CD105), a protein controlling placental angiogenesis [16], is a direct target of LXRalpha [17]. Soluble ENG is a marker of PE, and its serum concentration is tightly correlated with disease severity.

Single nucleotide polymorphisms (SNP) of the *NR1H3 *(LXRalpha) and *NR1H2 *(LXRbeta) genes have recently been associated with many metabolic indicators and conditions, including circulating total, LDL and HDL (High Density Lipoprotein)-cholesterol levels for *NR1H3 *[18-20], and type 2 diabetes mellitus and obesity for both *NR1H3 *and *NR1H2 *[21-24]. Based on these previous studies, we selected two SNPs (rs2279238 and rs7120118) in the *NR1H3 *gene, mapping to chromosome 11p11.2 and two SNPs (rs2695121 and rs35463555) in the *NR1H2 *gene, mapping to chromosome 19q13.3 for study. Rs2279238, rs2695121 and rs35463555 have been reported to be associated with obesity [22]; rs2279238 is also associated with risk factors for type 2 diabetes mellitus [23]. Finally, rs7120118 was chosen for study because it has been shown to be associated with HDL-cholesterol levels [20].

In a case-control study of 155 preeclamptic and 305 normal pregnancies based on a powerful high-resolution melting curve analysis technique, we showed that the *NR1H2 *polymorphism rs2695121 was strongly associated with PE. These findings suggest that further studies of the role of LXRbeta in the pathogenesis of this disease will be of interest. By contrast, we found no significant association between the *NR1H3 *SNPs (rs2279238 and rs7120118) and PE.

## Methods

### Ethics statement

This study was approved by the local ethics committee (CPP: *Comité de Protection des Personnes Sud Méditerranée III*). We recruited 155 patients between December 1997 and July 2002. Both cases and controls gave written informed consent for participation in the study. The clinical investigation was performed in accordance with the Helsinki Declaration and its amendments.

### Patients and controls

All the patients and controls were primiparous.

Patients were referred to the outpatient clinic of the Gynecology and Obstetrics Department or to the Hematology Laboratory, University Hospital of Nîmes, between December 1997 and July 2002, for thrombophilia screening and relevant investigations.

The inclusion criterion for the patients was preeclampsia during a natural pregnancy, defined as gravidic hypertension (systolic blood pressure [BP] > 140 mm Hg, diastolic BP > 90 mm Hg, a rise in systolic BP > 30 mm, or a rise in diastolic BP > 15 mm Hg on at least two measurements 6 hours apart) after 20 weeks, associated with significant proteinuria (> 300 mg/24 h). Thirty-six of the 155 preeclampsia patients had developed a severe form according to current definitions, before 34 weeks of gestation in nine cases. There were 11 cases of HELLP (hemolysis, elevated liver enzymes, low platelets) syndrome and two cases of eclampsia. We were unable to analyze severity subgroups, due to the small number and heterogeneity of the severe preeclampsia cases.

The 305 controls were women from the NOHA First Cohort, with uneventful pregnancies [25].

Data were collected for putative clinical predictors of preeclampsia: age, body mass index (BMI), smoking (defined as at least one cigarette per day), gravidity (evaluating the number of previous miscarriages), ethnicity, diabetes mellitus, pre-existing arterial hypertension (defined as the use of hypertensive medication, a systolic blood pressure of at least 140 mm Hg or a diastolic blood pressure of at least 90 mm Hg on two readings taken in a supine position 5 min apart, and on two separate occasions) and maternal history of preeclampsia.

### SNP genotyping

Genomic DNA was extracted from 10 ml of peripheral maternal whole blood collected into tubes containing EDTA (ethylenediaminetetraacetic acid) and sodium chloride, as described elsewhere [26]. The SNPs chosen for study were genotyped, as previously described [27]. Briefly, 10 ng of genomic DNA was subjected to PCR (polymerase chain reaction) amplification with LightCycler^® ^480 High-Resolution Melting Master Mix (Roche Diagnostics, Meylan, France), using a touchdown protocol as follows: initial denaturation at 95°C for 10 minutes then 50 cycles of denaturation at 95°C for 10 seconds; annealing at temperatures decreasing from 70°C to 60°C (step size: 0.5°C/cycle) for 20 seconds and elongation at 72°C for 20 seconds. High-resolution melting curve data were obtained by monitoring the decrease in fluorescence from 75°C to 95°C at a rate of 25 acquisitions per degree Celsius on a LightCycler^® ^480 apparatus (Roche Diagnostics). The sequences of the primers used for PCR are given in table 1. Fluorescence data were visualized by normalization, temperature-shifting and difference plotting, and were then analyzed with LightCycler^® ^Gene Scanning software (Roche Diagnostics). Melting curve analysis for rs2279238 and rs7120118 generated three distinct profiles corresponding to the three possible genotypes (frequent homozygous, heterozygous and rare homozygous, according to the NCBI SNP database (dbSNP; human build 132)). The identification of rare homozygous genotypes for rs2695121 and rs35463555 was made possible by diluting each sample with a known frequent homozygous genotype (2 ng) before PCR, thus generating a heterozygous profile, according to the kit manufacturer's recommendations. Genotyping results were confirmed by directly sequencing the PCR products of 10% of the samples with the primers used for PCR. PCR products were purified with the Agencourt AMPure XP kit (Beckman Coulter Genomics, Grenoble, France), according to the manufacturer's recommendations. Sequencing reactions were carried out with the BigDye^® ^Terminator v1.1 Cycle Sequencing Kit (Applied Biosystems, Courtaboeuf, France) and products were purified with the CleanSEQ kit (Beckman Coulter Genomics), according to the manufacturer's instructions. The purified products were then resolved on a 3130xl Genetic Analyzer (Applied Biosystems).

**Table 1:** Sequence primers used for high-resolution melting curve analyses

SNP (gene)	5'-3' sequences	Size of the amplicon (bp)
rs2279238 (*NR1H3*)	Fw: GAGAGCGTTGAAGCACTTTC	140
	Rev: GCTCAGAACATTGTAGTGGAAG	

rs7120118 (*NR1H3*)	Fw: CCTTTGGCACTTGTAGACTCAT	159
	Rev: GGAGGGGAGGAGACTTGAC	

rs35463555 (*NR1H2*)	Fw: CAGGAAGGAAGTGACAGAGACA	111
	Rev: CCATCGTTTGAGATTGTGGA	

rs2695121 (*NR1H2*)	Fw: GGCAGGTCTTGTTAGAAGGA	171
	Rev: AATACAGGGGATTGAGAGCC	

### Statistical analysis

Deviation from Hardy-Weinberg equilibrium (HWE) was assessed with DeFinetti software (T.F. Wienker and T.M. Strom, unpublished data, http://ihg.gsf.de/cgi-bin/hw/hwa1.pl). The frequencies of particular alleles and genotypes in cases and controls were assessed by χ^2 ^analysis (Statview software 5.0, SAS Institute). Analyses of the pairwise linkage disequilibrium (D' and r^2^) between the markers in each genomic region and haplotype analysis were carried out with Haploview 4.2 Software [28].

The putative predictors of preeclampsia -- the clinical factors listed above and the four polymorphisms studied -- were evaluated in women, initially by univariate analysis and then by multivariate logistic regression analysis. We calculated the corresponding crude odds ratio (cOR) and its 95% confidence interval (95% CI) and then the adjusted odds ratio (aOR) and its 95% CI. Variables for multivariate models were selected in a stepwise manner, beginning with all the variables for which *p *< 0.25 in univariate models as potential predictors, with final adjustment for all variables for which *p *< 0.25 in the univariate models.

## Results

### Characteristics of the participants

The characteristics of controls and patients at diagnosis are presented in table 2. There were no significant differences in age, ethnicity or percentage of smokers between the two groups. Body mass index, and the percentages of women with two previous spontaneous miscarriages, diabetes mellitus, pre-existing arterial hypertension or a maternal history of PE were significantly higher in the PE group than in controls.

**Table 2:** Clinical characteristics of controls and patients

Characteristics	Controls (N = 305)	Patients (N = 155)	*p*-value^a^	cOR (95% CI)	*p*-value^b^
Age	29 ± 7 (23-36)	30 ± 7 (23-37)	0.103	1.04 (0.99-1.09)	0.099

BMI	22.46 ± 2.60 (16.32-28.50)	22.80 ± 2.45 (17.74-29.40)	0.063	1.12 (1.02-1.22)	**0.021**

Smoking	13 (4.26)	3 (1.94)	0.309	0.44 (0.12-1.58)	0.210

Gravidity:			0.076		

1	259 (84.92)	121 (78.06)		1	

2	35 (11.48)	21 (13.55)		1.28 (0.72-2.30)	0.400

3	11 (3.61)	13 (8.39)		2.53 (1.10-5.81)	**0.029**

Ethnicity:			0.690		

Caucasian	291 (95.41)	145 (93.55)		1	

African	8 (2.62)	6 (3.87)		1.51 (0.51-4.42)	0.457

Asian	6 (1.97)	4 (2.58)		1.34 (0.37-4.82)	0.656

Diabetes Mellitus	4 (1.31)	7 (4.52)	0.071	3.56 (1.03-12.4)	**0.046**

Pre-existing arterial hypertension	8 (2.62)	12 (7.74)	0.021	3.12 (1.25-7.79)	**0.015**

Maternal PE antecedent	10 (3.28)	13 (8.39)	0.032	2.70 (1.16-6.31)	**0.022**

Age and BMI are presented as medians ± interquartile range and ranges are indicated in brackets. For nominal values, percentages are indicated in brackets. ^a^: Mann-Whitney test for continuous variables; ^b^: chi-square test for nominal variables. Abbreviations: BMI, body mass index; cOR, crude odds ratio; 95% CI, 95% confidence interval.

### Polymorphisms and risk of preeclampsia

Tables 3 and 4 show the allelic and genotypic frequencies, respectively, for cases and controls. Genotyping of the SNPs in the *NR1H3 *(LXRalpha) gene revealed that the T allele of rs2279238 was found in 14.10% of controls and 11.69% of cases; the C allele of rs7120118 was found in 25.74% of controls and 23.87% of cases. For the *NR1H2 *(LXRbeta) gene, the A allele of rs35463555 was found in 27.38% of controls and 32.90% of cases. The allelic frequencies of these three SNPs did not significantly differ between cases and controls. By contrast, the C allele of rs2695121 was found in 54.43% of controls and 64.84% of cases (*p *= 0.003). None of the distributions for the alleles and genotypes of the SNPs analyzed deviated from Hardy-Weinberg equilibrium in either cases or controls (data not shown).

**Table 3:** Allelic counts and frequencies of studied polymorphisms

SNP (gene)	SNP	Allele	Controls	Cases	*p*-value
LXRalpha (*NR1H3*)	rs2279238	C	524 (85.90)	272 (88.31)	
		
		T	86 (14.10)	36 (11.69)	0.310
	
	rs7120118	T	453 (74.26)	236 (76.13)	
		
		C	157 (25.74)	74 (23.87)	0.648

LXRbeta (*NR1H2*)	rs35463555	G	443 (72.62)	208 (67.10)	
		
		A	167 (27.38)	102 (32.90)	0.082
	
	rs2695121	T	278 (45.57)	109 (35.16)	
		
		C	332 (54.43)	201 (64.84)	**0.003**

The table presents total counts for each allele. The percentages of the alleles in each group (controls or cases) are indicated in brackets. A difference in allele distribution between the two groups was considered significant if the *p*-value was less than 0.05.

**Table 4:** Genotypic counts and frequencies of the studied polymorphisms

Gene	SNP	Genotype	Controls	Cases
LXRalpha (*NR1H3*)		C/C	225 (73.77)	119 (77.27)
		
	rs2279238	C/T	74 (24.26)	34 (22.08)
		
		T/T	6 (1.97)	1 (0.65)
	
		T/T	164 (53.77)	87 (56.13)
		
	rs7120118	T/C	125 (40.98)	62 (40.00)
		
		C/C	16 (5.25)	6 (3.87)

LXRbeta (*NR1H2*)		G/G	157 (51.48)	74 (47.74)
		
	rs35463555	G/A	129 (42.30)	60 (38.71)
		
		A/A	19 (6.23)	21 (13.55)
	
		T/T	70 (22.95)	21 (13.55)
		
	rs2695121	T/C	138 (45.25)	67 (43.23)
		
		C/C	97 (31.80)	67 (43.23)

The table presents total counts for each genotype. The percentages of the genotypes in each group (control or cases) are indicated in brackets.

We used these data to generate logistic regression models for each of the SNPs genotyped (Table 5). Univariate analyses indicated that the genotype distributions of *NR1H3 *(LXRalpha) SNPs (rs2279238 and rs7120118) did not differ significantly between cases and controls. The risk of PE in carriers of homozygous and heterozygous variant alleles for these two SNPs was similar to that in individuals homozygous for the wild-type alleles. Interestingly, both *NR1H2 *(LXRbeta) SNPs (rs35463555 and rs2695121) were found to be significantly associated with preeclampsia, which was more frequent in individuals homozygous for the mutated alleles. After adjustment for putative clinical predictors of preeclampsia -- pre-existing arterial hypertension, BMI, maternal history of PE, gravidity, diabetes mellitus, age and smoking -- *NR1H2 *SNP rs2695121 continued to be an independent risk factor for preeclampsia in multivariate logistic regression analysis (table 6). This significance was retained for the homozygous mutated allele C, unmasked for the heterozygous allele C and lost for the *NR1H2 *SNP rs35463555.

**Table 5:** Logistic regression analysis of the associations between LXRalpha or LXRbeta SNPs and preeclampsia (univariate analysis)

Gene	SNP	Genotype	cOR	95% CI	*p*-value
LXRalpha (*NR1H3*)	rs2279238	C/T	0.87	0.55-1.38	0.551
		
		T/T	0.32	0.04-2.65	0.288
	
	rs7120118	T/C	0.94	0.63-1.40	0.742
		
		C/C	0.71	0.27-1.87	0.485

LXRbeta (*NR1H2*)	rs35463555	G/A	0.99	0.65-1.49	0.949
		
		A/A	**2.35**	**1.19-4.63**	**0.014**
	
	rs2695121	T/C	1.62	0.92-2.86	0.097
		
		C/C	**2.30**	**1.29-4.11**	**0.005**

Abbreviations: cOR, crude odds ratio; 95% CI, 95% confidence interval.

**Table 6:** Logistic regression model of risk factors for PE (multivariate analysis)

Factor	aOR	95% CI	*p*-value
rs2695121-T/C	1.85	1.01-3.42	**0.049**

rs2695121-C/C	2.05	1.04-4.05	**0.039**

rs35463555-G/A	0.78	0.49-1.24	0.295

rs35463555-A/A	1.32	0.59-2.97	0.500

Pre-existing arterial hypertension	3.74	1.44-9.73	**0.007**

BMI	1.09	0.99-1.21	0.086

Maternal history of PE	2.35	0.95-5.80	0.065

Gravidity: 2	1.25	0.68-2.30	0.478

Gravidity: 3	2.89	1.22-6.88	**0.016**

Diabetes mellitus	3.35	0.90-12.5	0.073

Age	1.05	0.99-1.10	0.070

Smoking	0.66	0.18-2.46	0.535

Abbreviations: aOR, adjusted odds ratio; 95% CI, 95% confidence interval.

### Haplotype analysis

By studying the combinations of the two polymorphisms in each gene, we identified three common haplotypes for the *NR1H3 *(LXRalpha) gene and four for the *NR1H2 *(LXRbeta) gene (table 7). None of *NR1H3 *haplotypes was associated with PE. The two haplotypes of the *NR1H2 *gene (GT and AC) were associated with preeclampsia (*p *= 0.009 and *p *= 0.026, respectively). Haploblock analysis revealed that the two markers within each of the genes studied were in linkage disequilibrium (Additional file 1, Figure S1).

**Table 7:** Haplotypic prevalence of genotyped *NR1H3 *and *NR1H2 *in both groups

Gene	Haplotype	Controls (%)	Cases (%)	*p*-value
LXRalpha (*NR1H3*)	CT	0.742	0.755	0.687
	
	TC	0.141	0.109	0.183
	
	CC	0.117	0.129	0.579

LXRbeta (*NR1H2*)	GT	0.433	0.343	0.009
	
	GC	0.293	0.328	0.280
	
	AC	0.251	0.320	0.026
	
	AT	0.023	0.009	0.126

Haplotype frequencies were compared between cases and controls, in chi-squared tests.

## Discussion

This is the first study to investigate the association of the *NR1H2 *(LXRbeta) gene with preeclampsia. We report here a new genetic association between the SNP rs2695121, in the *NR1H2 *gene, and preeclampsia. By contrast, the two SNPs within the *NR1H3 *(LXRalpha) gene studied, rs2279238 and rs7120118, were not identified as independent risk factors for this disease.

**Figure 1:**
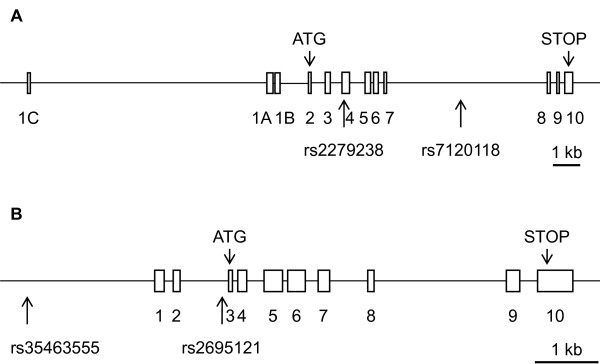
**Schematic representation of the *NR1H3 *(LXRalpha) and *NR1H2 *(LXRbeta) genes showing the position of the genotyped SNPs**. Rectangles indicate exons; lines indicate introns. Arrows under the schematic representation of the gene indicate the position of each genotyped SNP. Thick lines below the gene represent the 1 kb scale. A: schematic representation of the *NR1H3 *(LXRalpha) gene; B: schematic representation of the *NR1H2 *(LXRbeta) gene.

The four SNPs studied here were chosen on the basis of previously identified genetic associations with other physiological or physiopathological conditions. The rs2279238 SNP (NM_005693.2:c.297C > T (*p*. =)) is located within exon 4 of the *NR1H3 *gene (Figure 1A), but the nucleotide variation is synonymous, resulting in a protein sequence identical to that of the wild type. We also used four splice site prediction programs (SpliceSiteFinder-like, MaxEntScan, NNSPLICE and Human Splicing Finder) in Alamut 2.0 Software (Interactive Biosoftware, Mont-Saint-Aignan, France). These programs were unable to detect any clear splicing site modifications induced by the SNP within the gene. The SNP did not result in a predicted miRNA (microRNA) binding site (identified by MicroCosm Targets software) either. It is thus highly unlikely that this SNP was functional at the molecular level. Similarly, rs7120118 (NM_005693.2:c.988+2721T > C), which is located within an intron, would be unlikely to affect protein sequence and/or activity. The *NR1H2 *SNP rs2695121 (NM_007121.4:c.-19-103T > C), which was found to be associated with preeclampsia, is an intronic polymorphism that would therefore also be unlikely to affect protein function. This hypothesis was confirmed with the splice site prediction module of Alamut software. However, rs35463555 (NT_011109.16:g.23145898G > A), which was also found to be associated with PE in a univariate logistic regression model, is located within the promoter of the gene, 2 kb upstream from the transcription start site (Figure 1B). MatInspector analysis (Genomatic Software Suite, München, Germany) showed that this SNP was part of the core sequence of the NR2F (COUP-TFs) orphan nuclear receptor responsive element (data not shown). This program failed to identify this putative sequence when the A allele of the SNP was considered. An siRNA (small interfering RNA) directed against *NR2F1 *(COUP-TF1) has been shown to downregulate LXR expression and the expression of many LXR target genes in muscle cell cultures [29]. However, the authors of this previous study did not investigate the molecular mechanism underlying this regulation; no direct regulation of *NR1H2 *by COUP-TFs has thus been demonstrated to date. Moreover, the potential role of COUP-TFs in preeclampsia has never been investigated. Based on the results of this bioinformatic simulation, it may be interesting to evaluate the molecular effect of rs35463555 on the transcription of the gene encoding LXRbeta. These predictions suggest that the positive association between the *NR1H2 *rs2695121 SNP and preeclampsia may result from an indirect association rather than a direct effect of the polymorphism on protein function [30]. Thus, rs2695121 may be in linkage disequilibrium with an unobserved causal locus, which might even be located within another gene in the vicinity of *NR1H2*.

Despite the demonstration of an interesting association between the r2695121 polymorphism in *NR1H2 *(LXRbeta) and preeclampsia, this study was subject to several limitations. Univariate logistic regression analysis showed that both the SNPs within the *NR1H2 *gene tested were associated with preeclampsia. However, in a multivariate model including all the clinical cofactors and these two polymorphisms, only rs2695121 remained associated with PE. This association was strengthened by a significant adjusted link between the mutated homozygous but also heterozygous SNP and preeclampsia, but the lack of association between rs35463555 and the diseases warrants further investigation. It may result from there being too few samples in our study, but it could also reflect genetic linkage between the two polymorphisms. We calculated the linkage disequilibrium between the two polymorphisms in our genotyped population and we found evidence of genetic linkage between the two polymorphisms (D' = 0.85; r^2 ^= 0.21; Additional file 1, Figure S1), likely to account, at least in part, for the apparent lack of statistical power in our study.

Many risk factors for preeclampsia have been described. High body mass index, obesity and cholesterolemia are among the most frequently studied and are tightly linked to this disease [6,31]. High total and LDL-cholesterol levels have been identified as risk factors for preeclampsia. Our discovery of an association between a polymorphism of *NR1H2 *(LXRbeta) and PE is therefore of particular interest. Indeed, both LXRs are now widely considered to be major regulators of lipid homeostasis. In recent years, many studies in animals and *in vitro *models have demonstrated that these nuclear receptors modulate the expression of genes involved in various aspects of the control of cholesterol metabolism (For a review, see [7]). They may act on various pathways: inhibiting *de novo *cholesterol synthesis by downregulating key enzymes involved in its synthesis, stimulating the cellular efflux of cholesterol by upregulating ATP-binding cassette A1, G1 and G5/G8 (ABCA1, ABCG1 and ABCG5/G8) transporters and inducing the reverse transport of cholesterol by regulating apolipoprotein-encoding genes, such as *APOE *(apolipoprotein E). Circulating cholesterol has been widely described as a risk factor for preeclampsia, and some *APOE *alleles have been associated with PE [32]. Our work is thus consistent with recent findings of associations between polymorphisms in the genes encoding LXRalpha and LXRbeta and circulating total, LDL- and HDL-cholesterol concentrations [18-20] and obesity [22,24].

The physiopathological features of preeclampsia seem to result principally from placental ischemia and/or a maternal inflammatory response [1]. Many factors are involved in the development of this disease. In particular, the gene encoding cyclooxygenase-2 (COX-2) has been shown to be overexpressed in the vessels of women with PE [33], and circulating levels of proinflammatory cytokines, such as inteleukin-6 (IL-6), are higher in patients than in controls [34]. In addition to their well known cholesterolemia-lowering effects, LXRs have been implicated in immune processes. Their anti-inflammatory action was first described in 2003 in a study showing that their activation decreased the expression of many proinflammatory factors, including COX-2, iNOS (inducible nitric oxide synthase), IL-1beta (interleukin-1 beta) and IL-6 [35]. Thus, further studies on the role of LXRs in the immune processes associated with preeclampsia will be of particular interest.

Placental angiogenesis also makes a major contribution to preeclampsia. Indeed, defects in the development, differentiation and angiogenesis of the placenta may lead to PE [12]. Defects in signaling by angiogenic factors, such as vascular endothelial growth factor (VEGF), are also frequently observed. Circulating levels of sFLT-1 (soluble VEGF receptor 1) and sENG (soluble endoglin) are high in patients suffering from PE, and may contribute to the pathogenesis of the disease [16,32]. Interestingly, *VEGF *is a direct target gene of both LXRs [36]. We have also previously reported that *ENG *is a direct target of LXRalpha in human placental cell cultures [17]. Direct effects of liver X receptors on the placenta, such as trophoblast invasion [15,37] and syncytialization [38], have recently been suggested. One recent study revealed that the expression of *NR1H3*, *NR1H2 *and their target gene *ABCA1 *in JAR cells is stimulated under conditions of low oxygen concentration, mimicking the conditions occurring in preeclampsia [14]. The authors hypothesized that these deregulations might affect cholesterol transport between the mother and the fetus. Overall, these data provide evidence for a functional role for LXRs in the maintenance of placental homeostasis.

Human genetic studies have recently demonstrated associations between polymorphisms of the *NR1H3 *(LXRalpha) gene and circulating total, LDL- and HDL-cholesterol [18-20]. A role for LXRalpha in the pathogenesis of preeclampsia is also supported by our previous work showing that LXRalpha but not LXRbeta induced the promoter of *ENG *gene [17]. Our work reveals that the *NR1H2 *(LXRbeta) SNP rs2695121 is a risk factor for preeclampsia. The lack of association between the two tested *NR1H3 *SNPs and preeclampsia in our work is quite surprising. However, we limited our study to only two polymorphisms of *NR1H3 *gene, which had already been reported to be associated with physiological and/or pathological conditions. We cannot, on the basis of our results, exclude the possibility that *NR1H3 *is involved in the pathogenesis of preeclampsia, for two major reasons: the statistical power of this study may simply have been too low to pick up a significant association. Moreover, other SNPs, located within the same gene and not in strong linkage disequilibrium with rs2279238 and rs7120118, may be associated with the disease. Indeed, using Tagger implemented in Haploview software with an aggressive tagging method (r^2 ^threshold = 0.8) [39], we showed that our two SNPs covered 91% of the haplotypic variability of the *NR1H3 *gene (including 2 kb on either side of the gene; mean max r^2 ^= 0.97). However, as they did not account for all the variability, it is possible that the absence of association was due to missed haplotypes. Additional studies including more *NR1H3 *SNPs and/or more samples are therefore required to rule out definitively the existence of an association between this gene and the disease. The two polymorphisms of the *NR1H2 *gene studied (rs2695121 and rs35463555) have not yet been genotyped in the CEU+TSI populations of the Hapmap project. However, only three SNPs within this gene region have been described in the Hapmap project. These three SNPs are in strong linkage disequilibrium, defining a unique haploblock. As the two TagSNPs proposed by Tagger have never been associated with any physiological or physiopathological condition, we preferred to investigate two potentially functional SNPs described in previous studies, despite the possible lack of haplotypic variability coverage. A deeper replication study, including a larger number of polymorphisms, may facilitate definition of the contribution of *NR1H2 *to the pathogenesis of preeclampsia. In parallel, it will be interesting to determine the concentration of circulating endoglin in our cohort of patients, to identify possible associations with *NR1H2 *genotypes.

## Conclusions

In conclusion, we demonstrate here, for the first time, an association between a polymorphism of the *NR1H2 *(LXRbeta) gene and preeclampsia. However, caution is required in the clinical interpretation of risk factors [40]. The results of this retrospective pilot study will therefore require confirmation in a secondary prospective study on a set of patients, in which it should be possible to assess the association between the SNPs and disease severity. In addition to improving our understanding of the molecular physiopathological features of preeclampsia, this study opens up new possibilities for investigation. In particular, it will be particularly interesting to determine whether LXRbeta could serve as a new marker of the disease. Moreover, as this nuclear receptor is an inducible transcription factor, an understanding of its role in preeclampsia could guide the development of new ligands for the treatment of this disease.

## List of abbreviations used

95% CI: 95% confidence interval; ABC: ATP-binding cassette; APO: apolipoprotein; BP: blood pressure; COUP-TF: chicken ovalbumin upstream promoter transcription factor; COX-2: cyclooxygenase-2; HDL: high-density lipoprotein; HELLP: hemolysis: elevated liver enzymes: low platelets; HWE: Hardy-Weinberg equilibrium; IL-6: interleukin-6; iNOS: inducible nitric oxide synthase; LDL: low-density lipoprotein; LXR: liver X receptor; ND: not determinable; OR: odds ratio; PE: preeclampsia; RXR: retinoid × receptor; sENG: soluble endoglin; sFLT-1: soluble VEGF receptor 1; SNP: single nucleotide polymorphism; VEGF: vascular endothelial growth factor.

## Competing interests

The authors declare that they have no competing interests.

## Authors' contributions

KM, EM, SL, JCG conceived and designed the experiments. KM, EM, JCG acquired the data. KM, JCG analyzed and interpreted the data. EM, AP, AE, SB, JPB^4^, JPB^1 ^contributed to experimental design and revised the manuscript critically. KM, SL, JCG wrote the paper. All authors read and approved the final version of the manuscript.

## Pre-publication history

The pre-publication history for this paper can be accessed here:

http://www.biomedcentral.com/1471-2350/12/145/prepub

## Supplementary Material

Additional file 1 **Figure S1: Linkage disequilibrium between *NR1H3 *and *NR1H2 *SNPs**. The r^2 ^values are shown. D' was 0.97 and 0.85 between SNPs within the *NR1H3 *(LXRalpha) and *NR1H2 *(LXRbeta) genes, respectively. Click here for file
