# Supplementary figures and images for: A common polymorphism in NR1H2 (LXRbeta) is associated with preeclampsia
Source: BMC Med Genet. 2011 Oct 26;12:145. doi: 10.1186/1471-2350-12-145 (PMC3214159; doi:10.1186/1471-2350-12-145)

## Slide 1
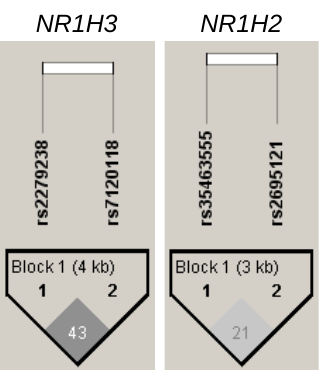

NR1H3
NR1H2

Supplement: Additional file 1 — Figure S1: Linkage disequilibrium between NR1H3 and NR1H2 SNPs. The r2 values are shown. D' was 0.97 and 0.85 between SNPs within the NR1H3 (LXRalpha) and NR1H2 (LXRbeta) genes, respectively. [file 1471-2350-12-145-S1.PPT]
